# Compliance in oxygen saturation targeting in preterm infants: a systematic review

**DOI:** 10.1007/s00431-015-2643-0

**Published:** 2015-10-14

**Authors:** Henriëtte A. van Zanten, Ratna N. G. B. Tan, Agnes van den Hoogen, Enrico Lopriore, Arjan B. te Pas

**Affiliations:** Department of Pediatrics, Division of Neonatology, Leiden University Medical Center, J6-S, PO Box 9600, 2300 RC Leiden, The Netherlands; Utrecht Medical Center, Utrecht, The Netherlands

**Keywords:** Preterm infant, Targeting oxygen, Compliance, Alarm limits, Hyperoxaemia, Hypoxaemia, Automated oxygen

## Abstract

During oxygen therapy in preterm infants, targeting oxygen saturation is important for avoiding hypoxaemia and hyperoxaemia, but this can be very difficult and challenging for neonatal nurses. We systematically reviewed the qualitative and quantitative studies investigating the compliance in targeting oxygen saturation in preterm infants and factors that influence this compliance. We searched PubMed, Embase, Web of Science, Cochrane, CINAHL and ScienceDirect from 2000 to January 2015. Sixteen studies were selected, which involved a total of 2935 nurses and 574 infants. The studies varied in methodology, and we have therefore used a narrative account to describe the data. The main finding is that there is a low compliance in oxygen targeting; the upper alarm limits are inappropriately set, and maintaining the saturation (SpO_2_) below the upper limit presented particular difficulties. Although there is little data available, the studies indicate that training, titration protocols and decreasing workload could improve awareness and compliance. Automated oxygen regulations have been shown to increase the time that SpO_2_ is within the target range.

*Conclusion*: The compliance in targeting oxygen during oxygen therapy in preterm infants is low, especially in maintaining the SpO_2_ below the upper limit.

**Table:** 

**What is Known:** • *The use of oxygen in preterm infants is vital, but the optimal strategy remains controversial.* • *Targeting SpO*_*2*_ *during oxygen therapy in preterm infants has been shown to reduce mortality and morbidity.*
**What is New**: • *Review of the literature showed that the compliance in targeting SpO*_*2*_ *and alarm settings is low.* • *Creating awareness of risks of oxygen therapy and benefits in targeting, decreasing nurse/patient ratio and automated oxygen therapy could increase compliance.*

## Introduction

Supplemental oxygen is often administered to preterm infants for hypoxemic episodes during respiratory distress or apnoeas. It is important to prevent hypoxaemia (defined as a decrease in arterial blood saturation (SpO_2_) of ≤80 % for ≥10 s), as frequent episodes could lead to an increased risk of morbidities, including retinopathy of prematurity (ROP), impaired growth, longer term cardio-respiratory instability and adverse neurodevelopmental outcome [12, 15, 30]. In extreme cases, it can even lead to death [12, 15]. Hyperoxaemia (SpO_2_ of >95 % for ≥10 s) also needs to be prevented, as administering supplemental oxygen can potentially lead to high oxygen levels. High concentration of oxygen is toxic to living cells and is known to be an important pathogenic factor for bronchopulmonary dysplasia (BPD) and ROP [31] and is correlated with cerebral palsy [3].

Pulse oximetry (PO) is most commonly used for continuous monitoring of oxygen saturation (SpO_2_) in a non-invasive manner [26]. To prevent hypoxaemia and hyperoxaemia, nurses usually titrate oxygen manually to maintain SpO_2_ between the prescribed target ranges. However, maintaining the SpO_2_ within this range can be challenging, and compliance—defined as the nurse’s behaviour that follows the clinical guidelines—[13] is influenced by several factors [40]. This compliance is important, as it can largely influence the effect of a certain SpO_2_ target range. The optimal range of SpO_2_ for preterm infants remains undefined, but recent trials have shown that aiming for 91–95 % has decreased mortality but increased incidence of ROP [36]. However, in these trials, oxygen was titrated manually, which caused a large overlap in the distribution of SpO_2_ between the two groups and may have decreased the observed differences in outcome.

Although comparison of SpO_2_ target ranges has been subject to systemic review [19], a review in the compliance in SpO_2_ target ranges is not available but equally important as which target range is optimal. The purpose of this study is to systematically review the available literature in compliance—and the factors influencing this compliance—in targeting SpO_2_ in preterm infants.

## Methods

**Fig. 1:**
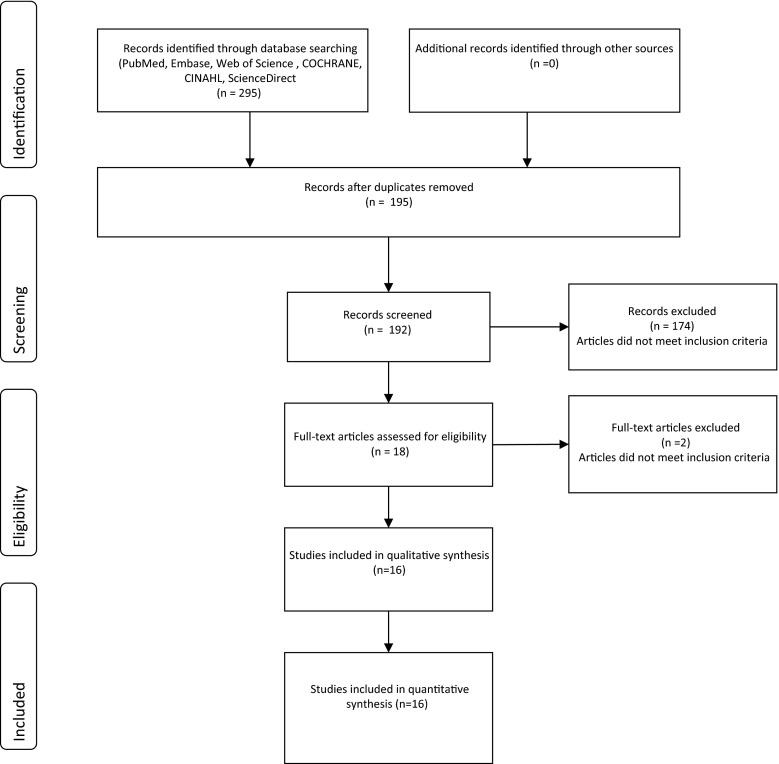
Flow diagram selection studies

We performed a systematic review, following PRISMA guidelines where possible (Fig. 1) [28]. The aim of the PRISMA statement is to help authors improve the reporting of systematic reviews and meta-analyses, which made it a particularly useful framework for this report. Eligible studies were identified by searching online databases from January 1965 to January 2015 in PubMed, Embase, Web of Science, Cochrane, CINAHL and ScienceDirect (keywords in Table 1). After selecting the eligible studies, we manually searched the reference lists of the selected studies to identify additional references. The criteria for inclusion limited the selection to articles published in English or Dutch which referred to preterm infants, (nursing) compliance, SpO_2_ monitoring by PO and targeting oxygen saturation during NICU admission. Both qualitative and quantitative designs were included, but publications that were not primary research studies, i.e. letters, abstracts, reviews and editorials, were not (Fig. 1).

**Table 1:** Keywords in different databases

Database	Keywords (including MeSH) terms
PubMed	Hyperoxia^a^, Hyperoxia*, hyperoxygenation, Hyperoxias, Hyperoxie, Hyperoxic, Hyperox*, hyperoxemic episode, hyperoxemic episodes, hypoxia, hypox*, hypoxemic episode, hypoxemic episodes, cyanosis cyanoses pulse oximetry, pulse oximeter, pulse oximeters, Infant^a^, Premature^a^, prematurity, prematur*, Pre-mature, pre-maturity, preterm, preterm*, low birth weight infant, low birth weight infants, Oxygen Inhalation Therapy^a^, Hyperbaric Oxygenation^a^, Oxygen/administration and dosage^a^, oxygen/therapeutic use + Oxygen/therapy^a^, Oxygen/Consumption^a^, oxygen consumption, oxygen, oxygenation, FiO2, FiO 2, FiO(2), FiO, increas*, fraction* exposure*, increase oxygen, increased oxygen, oxygen supplementation, oxygen therapy, supplemental oxygen, Automated closed loop control, FIO2 automatic, FIO2 adjustment closed-loop, FIO2 control, Oxygen Inhalation Therapy/instrumentation^a^, Respiration, Artificial/instrumentation^a^ complia*, nursing compliance, Adherence, adher*, Guideline Adherence^a^, Advance Directive Adherence^a^, Goals^a^, nursing procedures
CINAHL	Hyperoxia, hyperoxias, Hyperoxia* hyperoxygenation, hyperoxie, hyperoxic, hyperox* cyanosis, cyanoses, hypoxia*, pulse oximetry, pulse oximeter, pulse oximeters, prematur*, Prematurity, pre-mature, pre-maturity, preterm, preterm*, pre-term, low birth weight infant, low birth weight infants, Oxygen*, FiO2, FiO 2, FiO(2), FiO, increas*, fraction, fractions, fraction*, exposure, exposures, exposure*, increase oxygen, increased oxygen, oxygen supplementation, supplemental oxygen, oxygen saturation, oxygen administration, oxygen therapy, Automated closed loop control, FIO2 automatic, FIO2 adjustment closed-loop, FIO2 control, compliance, complia*, nursing compliance, Adherence, adher*
Web of Science	Hyperoxia, Hyperoxias, Hyperoxia*, Hyperoxie, Hyperoxic, hyperoxygenation, Hyperox*, cyanosis, cyanoses pulse oximetry, pulse oximeter, pulse oximeters, hypoxia*, hypoxemic episodes, hyperoxemic episodes, hypoxemic episode, hyperoxemic episode, cyanosis, cyanoses, premature, Prematurity, prematur*, pre-mature, pre-maturity, preterm, preterm*, pre-term, elbw infant*, low birth weight infant*, Oxygen*, FiO2, FiO 2, FiO(2), FiO, increas*, fraction, fractions, fraction*, exposure, exposures, exposure*, increase oxygen, increased oxygen, oxygen supplementation, supplemental oxygen, oxygen saturation, oxygen administration, oxygen therapy, Automated closed loop control, FIO2 automatic, FIO2 adjustment closed-loop, FIO2 control, compliance, complia*, nursing compliance, Adherence OR adher*
Embase	Hyperoxia/, pulse oximetry/, exp Hypoxia/, Hyperoxia, Hyperoxias, Hyperoxia*, Hyperoxie, Hyperoxic, hyperoxygenation, Hyperox*, pulse oximetry, pulse oximeter, pulse oximeters, hypoxia, hypoxemic episodes, “hyperoxemic episodes” hypoxemic episode, hyperoxemic episode, cyanosis/, cyanosis, cyanoses, prematurity/, premature, Prematurity, prematur*, pre-mature, pre-maturity, preterm, preterm*, pre-term, low birth weight infant, low birth weight infants, Oxygen*, FiO2, FiO 2, FiO(2), FiO) increas*, fraction, fractions, fraction*, exposure, exposures, exposure*, increase oxygen, increased oxygen, oxygen supplementation, supplemental oxygen, oxygen saturation, oxygen administration, oxygen therapy, exp oxygen therapy/, oxygen saturation/, Automated closed loop control, FIO2 automatic, FIO2 adjustment closed-loop, FIO2 control, oxygen delivery device/, exp *patient compliance/ compliance, complia*, nursing compliance, Adherence, adher* “nursing procedures”
ScienceDirect	Hyperoxia, Hyperoxias, Hyperoxia*, Hyperoxie, Hyperoxic, hyperoxygenation, Hyperox*, pulse oximetry, pulse oximeter, pulse oximeters, hypoxia*, cyanosis, cyanoses, premature, Prematurity, prematur*, pre-mature, pre-maturity, preterm, preterm*, pre-term, low birth weight infant, low birth weight infants, Oxygen*, FiO2, FiO 2, FiO(2), FiO, increas*, fraction, fractions, fraction*, exposure, exposures, exposure*, increase oxygen, increased oxygen, oxygen supplementation, supplemental oxygen, oxygen saturation, oxygen administration, oxygen therapy, Automated closed loop control, FIO2 automatic, FIO2 adjustment closed-loop, FIO2 control, compliance OR complia* OR nursing compliance OR Adherence OR adher*
Cochrane	Hyperoxia, Hyperoxias, Hyperoxia*, Hyperoxie, Hyperoxic, hyperoxygenation, Hyperox*, pulse oximetry, pulse oximeter, pulse oximeters, hypoxia*, premature, Prematurity, prematur*, pre-mature, pre-maturity, preterm, preterm*, pre-term, low birth weight infant, low birth weight infants, Oxygen*, FiO2, FiO 2, FiO(2), FiO, increas*, fraction, fractions, fraction*, exposure, exposures, exposure*, increase oxygen, increased oxygen, oxygen supplementation, supplemental oxygen, oxygen saturation, oxygen administration, oxygen therapy, Automated closed loop control, FIO2 automatic, FIO2 adjustment closed-loop, FIO2 control, compliance, complia*, nursing compliance, Adherence, adher*

^a^Keywords that were MeSH terms

Three authors (HvZ, RT, AH) independently graded the selected studies using the QualSyst tool for quantitative and qualitative studies [21]. In case of disagreement, consensus was reached through discussion or consultation of a fourth co-author (AtP). The QualSyst tool for quantitative studies is a validated generic checklist consisting 14 items with scores from zero to two and the possibility to score ‘not applicable’. Items rated not applicable were excluded from the calculation of the summary score. The maximum total score is 28. The summary score was calculated by summing the total score obtained across the relevant items and dividing that by the total possible score.

The QualSyst tool for qualitative studies is a validated generic checklist consisting of ten items with scores from zero to two, with the maximum total score of 20. A summary score was calculated for each study by summing the total score across the ten items and dividing them by the total possible score of 20 [21].

Data from selected studies were extracted using a data extraction form. The following study characteristics were extracted: author, year, design, sample, time points, length of measurement, target range and key results.

## Results

Sixteen articles met the inclusion criteria for this review (Fig. 1), detailing studies that included a total of 574 infants and 2935 nurses. Fourteen of these studies used a quantitative design [1, 7–10, 17, 18, 22, 25, 27, 34, 38, 39, 41] while the remaining two used qualitative methods [2, 29]. There was no homogeneity in the study designs, so pooling the data for meta-analysis was not possible. We therefore discuss the studies and their results using a narrative format organized under thematic headings and summarized in tables.

## Quality assessment

The studies varied in quality, but none was excluded because of low-quality scores. One observed weakness was the lack of power analysis in four of the studies [10, 17, 27, 39], and all studies were unclear in the reasoning behind the timing and duration of SpO_2_ data collection [1, 2, 7, 10, 17, 18, 22, 25, 27, 29, 34, 38, 39, 41] (Tables 2 and 3).

**Table 2:** Quality appraisal of included quantitative studies

Quality assessment quantitative studies															
Studies	Question	2. Study design	3. Selection	4. Subject characteristics	5. Random allocation	6. Blinding investigator	7. Blinding subjects	8. Outcome	9. Sample size	10. Analytic methods	11. Estimate of variance	12. Confounding	13. Results	14. Conclusion	Summary score
Claure, N. et al. (2001)	1	1	1	2	1	0	n/a	1	n/a	2	2	1	1	1	14/24 = 0.58
Claure, N. et al. (2009)	1	1	1	2	1	0	n/a	1	2	2	2	1	1	1	16/26 = 0.62
Claure, N. et al. (2011)	2	2	2	2	1	0	0	2	2	2	2	1	2	2	22/28 = 0.79
Clucas, L. et al. (2007)	2	2	1	2	0	0	0	2	0	2	2	1	2	2	18/28 = 0.64
Hagadorn, J.I. et al. 2006)	2	2	1	2	1	0	0	1	0	2	2	1	1	1	16/28 = 0.57
Laptook, A.R. et al. (2006)	1	1	1	2	0	0	0	1	2	2	2	1	1	1	15/28 = 0.54
Mills, B.A. et al. (2010)	2	2	1	2	1	0	0	1	0	2	2	1	1	2	17/28 = 0.61
Sink, D.W. et al. (2011)	2	1	1	1	0	0	n/a	1	n/a	2	0	1	1	1	11/24 = 0.46
Urschitz, M.S. et al. (2004)	2	2	2	2	2	0	0	1	2	2	2	1	2	2	22/28 = 0.79
Van der Eijk, A.C. et al. (2012)	1	2	2	2	0	0	0	1	0	2	1	1	1	1	14/28 = 0.5
Zapata, J. et al. (2014)	2	2	2	2	2	0	0	2	1	2	2	1	2	2	22/28 = 0.79
Lim, K. et al. (2014)	2	2	2	2	n/a	n/a	n/a	2	n/a	2	2	2	2	2	20/20 = 1
Arawiran, J. et al. (2014)	2	2	2	2	n/a	n/a	n/a	1	1	2	2	1	2	1	18/22 = 0.82
Hallenberger, A. et al. (2014)	2	2	2	2	2	0	0	2	2	2	2	1	1	1	21/28 = 0.75

2 = yes; 1 = partial; 0 = no; n/a = not applicable

**Table 3:** Quality appraisal of included qualitative studies

Quality assessment qualitative studies											
Studies	1. Question/objective	2. Study design	3. Context	4. Theoretical framework	5. Sampling strategy	6. Data collection	7. Data analysis	8. Verification procedure	9. Conclusion	10. Reflexivity	Summary score
Nghiem, T.H. et al. (2008)	2	2	2	2	1	2	2	0	1	2	16/20 = 0.8
Armbruster, J. et al. (2010)	1	2	2	2	2	2	0	0	2	2	15/20 = 0.75

2 = yes; 1 = partial; 0 = no

## Study designs

The designs of the quantitative studies varied and were composed of the following: one efficacy study [9], two pilot clinical trials [6, 41], three randomized clinical trials [7, 18, 38] and eight observational studies, of which six had a prospective design [1, 10, 17, 22, 25, 27] and two were retrospective [34, 39] (Table 4). Both qualitative studies employed a descriptive design [2, 29] (Table 4).

**Table 4:** Summery of included studies

Author	Year	Design	Study objects	Timing of measurement	Target range	Key results
Armbruster, J. et al.	2010	Qualitative study with individual open-ended interviews	41 nurses	First 3 days of life while infants were receiving supplemental oxygen	88–92 %	Saturations of infants in the Canadian Oxygen Trial (COT) study were in the intended range in 68–79 % of time. Nurses identified education, prompt response to alarm limits and a favourable patient to staff ratio as important determinants of good compliance
Claure, N. et al.	2009	Pilot clinical trial	16 premature infants, GA 24.9 ± 1.4 weeks receiving mechanical ventilation and FiO_2_	4-h period with FiO_2_ adjustment by clinical staff members (manual) and 4-h period with automated FiO_2_ adjustments (automated) PNA 33 days (SD ± 15)	88–95 %	In automated mode: • % of time within SpO_2_ target range was 58 % • % of time that SpO_2_ >95 % was 9 % • % of time that SpO_2_ <88 % was 33 % In manual mode: • % of time within SpO_2_ target range was 42 % • % of time that SpO_2_ >95 % was 31 % • % of time that SpO_2_ <88 % was 27 %
Claure, N. et al.	2001	Efficacy study	14 infants, GA 25 weeks (SD ±1.6) receiving mechanical ventilation and FiO_2_	2 h in manual FiO_2_ mode and 2 h in automatic FiO_2_ mode in random sequence. PNA 26 days (SD ± 11)	88–96 %	In automatic FiO_2_ mode • % of time within SpO_2_ target range was 74.9 % • The percentage of time that saturations were <88 % was 16.5 % and • >96 % in 9.9 % of the time. In manual FiO_2_ mode • % of time within SpO_2_ target range was 66.3 % • The percentage of time that saturations <88 % was 18.7 % and • >96 % in 14.9 % of time.
Claure, N. et al.	2011	Clinical trial	32 premature infants GA 25 weeks (24–27) receiving mechanical ventilation and FiO_2_	24-h period with FiO_2_ adjustment by clinical staff members (manual) and 24-h period with automated FiO_2_ adjustments (automated) PNA 27 days (range 17–36)	87–93 %	In automated mode: • % of time within SpO_2_ target range was 40 % • % of time that SpO_2_ >93 % was 28 % • % of time that SpO_2_ <87 % was 32 % In manual mode: • % of time within SpO_2_ target range was 32 % • % of time that SpO_2_ >93 % was 43 % • % of time that SpO_2_ <87 % was 23 %
Clucas, L. et al.	2007	Prospective cohort study	80 infants with receiving supplemental oxygen Mean GA of 28.4 weeks (SD ±2.4) 1073 lower and upper alarm limit values	Daily during weekdays, when the infant was on oxygen until discharge PNA 5 days (IQR 2–34.5)	88–92 %	The lower alarm limit was set correctly in 91.1 % of the time, 6.3 % was set lower, and 2.7 % was set higher than intended; upper alarm limit was set correctly in 23.3 % of the time, 0.2 % was set lower, and 76.5 % was set higher than intended.
Hagadorn, J.I. et al.	2006	Prospective multicentre cohort study	84 infants GA 26.3 Median: (29.4–27.4) 14 centres from three counties 307 monitor periods of median duration of 67.3 h	Saturation for 72 h each week for the first 4 weeks of life	Centre-specific intended TR 92–96 % 90–95 % 88–95 % 88–97 % 88–92 % 87–94 % 92–96 % 90–96 % 85–98 % 88–94 % 85–94 % 88–92 % 83–93 %	Overall, infants spent 16 % below intended range and 36 % above their NICU’s intended range
Laptook, A.R. et al.	2006	Prospective observational study	Group 1: 23 infants GA 27 weeks (±2) receiving continuous supplemental oxygen (with or without ventilator) Group 2: 49 infants, GA 26 weeks (±2) receiving continuous supplemental oxygen (with or without ventilator)	24 h of data twice a month during 6 months when the author was available PNA group 1, 23 days (±21) PNA group 2, 23 days (±19)	Group 1: target range 90–95 %, Group 2: target range 88–94 %	Group 1: SpO_2_ values were under target range in 26.9 % and above the target range in 15.4 % of time Group 2: SpO_2_ values were under target range in 26.6 % and above the target range in 14.0 % of time
Mills, B.A. et al.	2010	Prospective cohort study	56 infants mean GA 26.7 weeks (SD 2.0) receiving supplemental oxygen 22 infants in BOOST II trial Number of recordings = 454	Daily during weekdays, when the infant was on oxygen until discharge	88–92 %	Lower alarm limits: In BOOST II trial; 94.2 % was set correctly; Not in BOOST II; 87.3 % was set correctly. Upper alarm limits: In BOOST II trial; 79.8 % was set correctly; Not in BOOST II; 28.8 % was set correctly
Nghiem, T.H. et al.	2008	Survey	59 NICUs 2805 nurses who submitted surveys	First 4 weeks of life of preterm infants	68 % of included NICUs, had policy specified SpO_2_ target limits; not exactly defined	Of 1957 nurses at NICUs with policies, 64 % of nurses were aware that policy for SpO_2_ was present in their NICU. 715 (37 %) nurses correctly identified the SpO_2_ limits specified by their NICU policy
Sink, D.W. et al.	2011	Retrospective observational study	14 infants GA <26.6 weeks (SD ± 1.6) with oximeter data 87 nurses	Every 2 s during routine bedside oximetry monitoring PNA 31.6 weeks (mean range 24.1–40.7 weeks)	85–92 %	Oxygen saturations in infants <28 GA were 61 % above intended range and 6 % under de intended range. Infants of 28–31 weeks of gestation were 70 % above intended range and 7 % under de intended range. Hyperoxic time increased from 48 to 71 % with assignment of a second patient to the infant’s nurse and to 82 % with assignment of a third patient to the infant’s nurse
Urschitz, M.S. et al.	2004	Randomized controlled clinical trial (validation and efficacy trial)	Validation trial: 12 preterm infants GA; median (IQR) 24.5 (24–28) receiving ventilator support and FiO_2_ Efficacy trial; 12 preterm infants GA; median (IQR) 25.5 (24–33) receiving ventilator support and FiO_2_	1 day during five periods of different modes, 90 min in each mode • Baseline 1, • Routine manual control, • Optimal manual control, • Closed-loop control, • Baseline 2 Validation trial: PNA 21 days (median range 8–57) Efficacy trial: PNA 20.5 days (median range 4–78)	87–96 %	Validation trial: % of time within SpO_2_ target range was • Baseline 1, 75.3 % • Routine manual control, 79.7 % • Optimal manual control, 85.8 % • Closed-loop control, 82.1 % • Baseline 2, 79.4 % No information over hypoxaemic and hyperoxaemic periods Efficacy trial: % of time within SpO_2_ target range was • Baseline 1, 82.9 % • Routine manual control, 81.7 % • Optimal manual control, 91 % • Closed-loop control, 90.5 % • Baseline 2, 81.2 % Duration of hypoxic episodes • Baseline 1, 20.2 s (11.3 %) • Routine manual control, 19 s (10.7 %) • Optimal manual control, 16.4 s (9.2 %) • Closed-loop control, 12.4 s (7 %) • Baseline 2, 19.1 s (10.7 %) Duration of hyperoxic episodes • Baseline 1, 24.7 s (6.7 %) • Routine manual control, 19.3 s (5.2 %) • Optimal manual control, 16.4 s (5 %) • Closed-loop control, 10.1 s (2.7 %) • Baseline 2, 17.4 s (4.7 %)
Van der Eijk, A.C. et al.	2012	Observational cohort study	12 infants, median GA 26 2/7 weeks (range 24 2/7–28) with a need for supplemental oxygen	Recording started when FiO_2_ was >21 % in the first 2 weeks of life PNA 4 days (range 2–12)	88–94 %	SpO_2_ <88 % in 16 % of the time and >94 % in 30 % of the time
Zapata, J. et al.	2014	Pilot clinical trial	20 infants, mean GA 27.3 ± 1.7 vs 27.7 ± 1.7 weeks receiving supplemental oxygen by nasal cannula	12-h study period PNA 5–14 days	85–93 %	With automixer: • % of time within SpO_2_ target range was 58 % • % of time that SpO_2_ >95 % was 26.5 % • % of time that SpO_2_ <85 % was 14 % In manual routine care: • % of time within SpO_2_ target range was 33.7 % • % of time that SpO_2_ >95 % was 54.8 % • % of time that SpO_2_ <85 % was 11.5 %
Hallenberger, A. et al.	2014	Multicenter randomized controlled crossover clinical trial	34 infants median GA (range) 26.4 (23.0–35.3) receiving mechanical ventilation or nasal CPAP and supplemental oxygen	24-h period with routine manual control (RMC) and 24-h period with closed-loop automated control (CLAC) PNA 29.9 weeks (26.0–35.6) (median (range))	Four centre-specific TRs 90–95 % 80–92 % 83–93 % 85–94 %	In closed-loop automated control (CLAC): • % of time within SpO_2_ target range was 72.1 (13.6) (mean(SD)) • % of time that SpO_2_ above TR was 15.9 (1.9–34.8) (median (range)) • % of time that SpO_2_ below TR was 9.1 (1.9–24.2) (median (range)) In routine manual control (RMC): • % of time within SpO_2_ target range was 61.0 (15.2) (mean(SD)) • % of time that SpO_2_ above TR was 16.0 (0.0–60.0) (median (range)) • % of time that SpO_2_ below TR was 15.0 (0.5–39.6) (median (range))
Arawiran, J. et al.	2014	Prospective observational cohort study	71 premature infants GA <31 weeks Pre-intervention phase: 41 infants: 25 ± 1.6 weeks (mean ± SD) Postintervention phase: 30 infants: 25 ± 1.9 weeks (mean ± SD)	Study period from first day of life as long as they received supplemental oxygen or were taken off the Masimo monitors or reached 31 weeks postconceptual age, whichever occurred first	85–92 %	Pre-intervention phase: • Proportion of time spent per 12-h shift in which individual babies were (mean (%) ± SD (%)) • <70 % was 3.4 ± 2.6 • 70–74 % was 1.6 ± 1.3 • 75–79 % was 4.0 ± 2.9 • 80–84 % was 9.6 ± 5.63 • 85–92 % was 44.5 ± 14.4 • 93–100 % was 36.9 ± 17.2 Postintervention phase: • Proportion of time spent per 12-h shift in which individual babies were (mean (%) ± SD (%)) • <70 % was 3.3 ± 2.5 • 70–74 % was 1.6 ± 1.1 • 75–79 % was 3.9 ± 2.3 • 80–84 % was 8.9 ± 4.3 • 85–92 % was 40.4 ± 12.8 • 93–100 % was 41.9 ± 15.6
Lim, K. et al.	2014	Multicenter prospective observational cohort study	45 premature infants GA 30 (IQR 27–32 weeks) 2971 h receiving supplemental oxygen	Age at first recording was at day 1 (IQR 0–8 days)	88–92 %	Median proportion of time in % ((IQR)) • % of time within SpO_2_ target range was 31 % (19–39) • % of time that SpO_2_ >93 % was 59 % (36–74) • % of time that SpO_2_ <87 % was 9 % (4.3–18) More than one infant per nurse was associated with a greater frequency of significant hyperoxaemia (SpO_2_ >98 %) when infants were in supplemental oxygen, and a trend towards less normoxaemia

## TRs of SpO_2_

The lower limit of the target ranges (TRs) varied between studies from 80 to 92 % [17, 18], and upper limits of TR varied from 92 to 96 %, respectively [1, 9, 10, 17, 25, 27, 34] (Table 4).

## Time points and length of measurements

All studies were conducted in the period that the infants needed supplemental oxygen, but the starting time points and duration of data collection differed between studies. The starting time point varied between the first day of life [1, 2] and 33 days [8] (Table 4). In one study, the postnatal age was not described [27]. The duration of data collection also varied widely, the shortest covering only 4 h [9] and the longest the entire period between admission and discharge [10]. The data were collected continuously in eight studies [1, 7–9, 18, 34, 38, 39, 41] and intermittently in the remaining studies [10, 17, 22, 27] (Table 4).

## Compliance in TR

Twelve studies investigated how often SpO_2_ values were in or outside the TR, expressed as the percentage of monitored time [1, 7–9, 17, 18, 22, 25, 34, 38, 39, 41]. In a multicentre study, Hagadorn et al. observed that SpO_2_ was below, within or above TR in 16 (0–47 %), 48 (6–75 %) and 36 (5–90 %), respectively, of the monitored time [17]. Van der Eijk et al. reported similar values, finding that SpO_2_ was below TR for 16 % of the time and above it for 30 % [39]. In contrast, Lim et al. only studied infants receiving supplemental oxygen during CPAP and SpO_2_ was below TR for 9 % and above it for 58 % of the time [25].

## Education and training

Two studies demonstrated the impact of an educational program in targeting SpO_2_. Laptook et al. observed that training did not change the time that SpO_2_ was below (26.9 vs. 26.6 %; not significant (ns)) or above TR (15.4 vs. 14.0 %; ns) [22]. Interestingly, Arawiran et al. even observed that training had an adverse effect and that the time that SpO_2_ was within TR decreased after training (44.5 ± 14.4 vs 40.4 ± 12.8 %) with an increase in time above TR (from 36.9 ± 17.2 vs 41.9 ± 15.6 %) [1].

## Nurse/patient ratio

Sink et al. studied the influence of the nurse/patient ratio on compliance in SpO_2_ targeting. They observed that the proportion of time that SpO_2_ was below TR decreased from 0.06 to 0.03 and time above TR increased from 0.56 to 0.82 when a third or fourth patient was added to the nurse’s workload [34]. The high percentage of time above TR was probably due to the use of a lower upper limit (92 %) in comparison with other studies [7–9, 22, 38, 39]. Lim et al. also confirmed that more than one infant per nurse was associated with an increase in the time when SpO_2_ was above TR (Table 4) [25].

## Automated regulation of inspired oxygen

Six recent studies reported that, when compared to manual titration, the use of automated regulation of inspired oxygen increased the time that SpO_2_ spent within TR [7–9, 18, 38, 41]. In a multicenter crossover study of ventilated preterm infants, Claure et al. (2011) observed that the time that SpO_2_ was within TR increased significantly during the automated period compared with the manual period (40 % (14) vs 32 % (13) (mean (SD) *p* < 0.001). The time periods with SpO_2_ >93 % or >98 % were thus significantly reduced during the automated period [7]. Although most studies observed that the time that SpO_2_ was above TR decreased [7–9, 38, 41] while the time below TR increased [7, 8, 38, 41], Hallenberger et al. found different results. They observed no change in time above TR (16 (0.0–60) vs 15.9 (1.9–34.8) *p* = 0.108) during automatic control of inspired oxygen and, therefore, no difference with manual control [18] (Table 4).

## Compliance in alarm limit setting

Two studies investigated nursing compliance in setting the appropriate alarm limits for PO in preterm infants [10, 27]. The actual SpO_2_ values were not reported, but Clucas et al. observed that the lower and upper alarm limit was set correctly in 91 and 23 % of monitored time, respectively [10]. Mills et al. compared compliance in alarm settings of SpO_2_ according to whether or not infants participated in a trial. When infants were participating in the BOOST II trial, the lower and upper alarm limit for SpO_2_ was set correctly in 94 % (88–100 %) and 80 % (71–88 %) of the monitored time period. However, this decreased to 87 % (75–99 %) and 29 % (17–40 %) when infants were not participating in the trial [27] (Table 4).

## Nurses’ perception and awareness

Armbruster et al. interviewed nurses who stated that the following would improve their compliance: further education, prompt response to alarm limits, a favourable patient to staff ratio, root cause analyses at the bedside and high priority given to control oxygen therapy [2]. Nghiem et al. reported that 63 % of the nurses were aware of the local oxygen saturation guidelines and 57 % of them correctly identified the target limits specified by their NICU guidelines (Table 4) [29].

## Discussion

The wide variation in study methodologies made it necessary to use narrative reporting when discussing the results of this systematic review. Although the power of some of the studies was limited and the quality varied, all were considered eligible for inclusion. Moreover, they focused on different aspects of compliance in targeting SpO_2._ The design, TR of SpO_2_, time points and duration of each study differed.

The central finding is that compliance in targeting SpO_2_ was low, as were the alarm settings. All studies in compliance in oxygen targeting reported that maintaining the SpO_2_ below the upper limit was the most difficult to adhere to [1, 6, 7, 10, 17, 18, 25, 27, 34, 39, 41]. The analysis of the large clinical trials comparing lower- vs higher-oxygen-saturation TR was based on the intention to treat principle. However, the larger proportion of the SpO_2_ was either below or above the intended TR and there was also an overlap between the two TRs [4, 32, 36]. Although compliance was audited [27], it is possible that this has influenced the outcome of the trials. This underlines the importance in improving compliance in SpO_2_ targeting, as improved compliance could have influenced the results.

## According to the studies

Several factors may play a role in low compliance in targeting oxygen saturation: lack of awareness of the TR settings, limited knowledge of the effects of hypoxaemia and hyperoxaemia and an increased nurse/patient ratio [2, 23, 25, 29, 34]. Many caregivers were unaware of the appropriate SpO_2_ limits [29]. In addition, nurses tend to rely on subjective observations for oxygen titration, such as skin colour and chest excursions, as well as PO and blood gases [35]. So far, studies indicate that the effects of education and training in improving the compliance targeting SpO_2_ are disappointing [1, 23].

On the other hand, the use of automated FiO2 regulation, which eliminates the need for the nurses’ compliance, has been shown to improve the time that SpO_2_ remains within TR [7–9, 38, 41]. The increase in time within TR was small, but it is possible that the effect of automated FiO_2_ regulation has been underestimated. A Hawthorne effect could have increased the nurses’ compliance during the short study period, thus decreasing the difference between the manual and automated periods. The effectiveness of automated regulation on oxygenation variability, and whether this results in an improved outcome, remains to be investigated [5].

It has been suggested that the absence of a FiO_2_ titration protocol would lead to saturations which would frequently exceed or fall below the TR [24]. Manual adjustments of FiO_2_ can vary widely in frequency and step size, so standardization of these adjustments could decrease large fluctuations in SpO_2_ [39]. After implementing an oxygen titration protocol for reducing the incidence of severe ROP, Lau et al. observed that the period during which SpO_2_ was above TR decreased significantly [24].

Although fewer studies investigated this, compliance with alarm settings appeared to be low as well, especially the upper alarm limit [10, 27]. In addition, even when alarm limits are appropriately set, caregivers seem to have a preference for SpO_2_ close to the upper alarm limit [4, 20]. This was also demonstrated in the large trials comparing TR of SpO_2_ [36]. It is possible that caregivers are more accustomed to preventing hypoxaemia than hyperoxaemia. It is also possible that infants are more stable in SpO_2_ when kept at the higher end of the TR. A regular check of alarm limit settings each shift could increase awareness of this issue.

Educational programs on hyperoxaemia improved knowledge levels [11, 16] but did not lead to better compliance. Earlier research has shown that after education in risks related to hyperoxaemia, the nurses’ performance was still variable and only 51 % of nurses were successful in minimizing exposure of their infants to hyperoxaemia [37]. Nurses usually take care of more than one patient and perform multitasking [14], and an increased workload decreases their compliance in TR [25, 34]. Also, nurses frequently have to deal with alarms, but a large proportion of the alarms are false [33]. The common occurrence of false alarms or “cry wolf” phenomenon could lead to no or delayed response of caregivers.

The decision not to limit inclusion criteria in terms of study design and methodology led to a high level of variety within the chosen studies, necessitating a narrative review. The advantage of this method, however, is that it enabled us to have a complete overview of a range of different aspects related to compliance in SpO_2_ targeting. However, the review was restricted to recent studies published in English and Dutch; similar studies published in other languages may have been missed. In addition, the selection process was conducted by the first author only and selection bias could have occurred. However, in any case of doubt of including a publication, peers were approachable for discussion and were resolved by consensus to avoid bias in the selection process.

In conclusion, the main finding of this literature review is that there is a low compliance in SpO_2_ targeting and alarm settings during oxygen therapy in preterm infants, especially in maintaining the SpO_2_ below the upper limit and in setting the upper alarm limit. Although there is little data available, it is likely that training, titration protocols and decreasing the nurses’ workload could improve awareness and compliance. Automated oxygen regulations have been shown to increase the time that SpO_2_ remains within the TR. Improving the compliance in SpO_2_ targeting and automated control has the potential to improve the outcome in preterm infants. The effect of training, implementing protocols and automated oxygen regulators needs further investigation.

## Abbreviations

CPAP: Continuous positive airway pressure
NICU: Neonatal intensive care unit
PO: Pulse oximetry
SpO_2_: Oxygen saturation
TRs: Target ranges
